# A case of ventilatory impairment during per-oral endoscopic myotomy under general anesthesia

**DOI:** 10.1186/s40981-018-0160-7

**Published:** 2018-02-26

**Authors:** Takuya Okada, Shinichiro Izuta, Satoshi Mizobuchi

**Affiliations:** Division of Anesthesiology, Department of Surgery Related, Kobe University Graduate School of Medicine, 7-5-1 Kusunoki-cho, Chuo-ku, Kobe, 650-0017 Japan

**Keywords:** Per-oral endoscopic myotomy, Jackhammer esophagus, Gas-related complication, Ventilatory impairment

## Abstract

We report a case of unexpected ventilatory impairment that occurred during per-oral endoscopic myotomy (POEM) under general anesthesia. A 73-year-old woman underwent POEM for Jackhammer esophagus. The patient developed hypercarbia, pneumoperitoneum, and severe subcutaneous emphysema during the operation. Although she was treated with abdominal paracentesis, it became difficult to ventilate her lungs a few minutes later. We recommended the surgeons to interrupt the procedure and proposed repeating the abdominal paracentesis. Simultaneously, we switched to manual ventilation and waited for the subcutaneous emphysema to subside. Thereafter, her respiratory status gradually improved and the surgeons were able to continue the operation. We considered that the main reason for our patient’s severe ventilatory impairment was that the length of surgical dissection was longer than usual.

## Background

Per-oral endoscopic myotomy (POEM), which was first reported in 2008, is a relatively new endoscopic treatment for achalasia and other esophageal motility disorders, such as abnormal contraction of the esophageal muscle, presenting as spasm [1]. Several recent reports have demonstrated its efficacy and safety [2, 3]. Since then, POEM has been considered a good treatment option when the condition cannot be controlled by medication. However, since carbon dioxide is used during POEM, gas-related complications, such as subcutaneous emphysema, pneumoperitoneum, mediastinal emphysema, and pneumothorax, can occur, and these complications can sometimes become critical. However, there have been only few reports of serious respiratory complications during POEM [4]. Here, we report a case of unexpected ventilatory impairment during POEM under general anesthesia.

## Case presentation

The patient was a 73-year-old woman (height 155 cm, weight 52 kg) with a history of inhaler therapy and an oral steroid (prednisolone 5 mg) for asthma. She complained of progressively worsening dysphagia for 1 year, for which she was referred to our hospital for detailed examination. Evaluation at our hospital led to a diagnosis of Jackhammer esophagus, which is an esophageal motility disorder that is characterized by intense esophageal spasms involving all or most regions of the esophagus. Since her symptoms did not improve with medication, she was scheduled to undergo POEM under general anesthesia.

The operation was started in the afternoon due to operating room scheduling. To avoid pulmonary aspiration of regurgitated material under anesthesia, we instructed her to stop eating and drinking on the night before the operation. We did not administer premedication or perform esophagoscopy to evacuate esophageal contents.

On the day of the operation, the patient entered the operating room, and pulse oximetry (SpO_2_), noninvasive blood pressure, electrocardiography(ECG), capnometry, urine volume, and temperature monitoring were applied. General anesthesia was induced by rapid sequence induction with administration of remifentanil 100 μg, propofol 100 mg, and rocuronium 50 mg, and endotracheal intubation was uneventfully performed. Anesthesia was maintained with 1.5–2.0% sevoflurane in 40% oxygen with air. A continuous intravenous infusion of remifentanil (0.2–0.4 μg/kg/min) and intermittent administration of rocuronium were administered while the patient was mechanically ventilated. The respirator was set to deliver volume-controlled ventilation (VCV) with a tidal volume of 400 ml and respiratory rate of 14 breaths/min. Peak airway pressure (PAP) at the start of the operation was 15 cmH_2_O.

At the beginning of the operation, her vital signs remained stable. However, end-tidal CO_2_ (EtCO_2_) gradually increased from about 1 h after the start of surgery. At 1 h and 17 min after the start of the operation, EtCO_2_ increased to 60 mmHg and PAP reached 35 cmH_2_O. At that time, the ventilator was set for VCV, with a tidal volume of 500 ml and respiratory rate of 16 breaths/min. We identified pneumoperitoneum and subcutaneous emphysema by the presence of the characteristic crackling feel over her chest and abdomen. Immediately, the surgeons treated her with abdominal paracentesis; however, her PAP continued to increase despite the same volume-controlled ventilation settings. Although we changed from mechanical to manual ventilation, it became more difficult to ventilate her lungs and her tidal volume decreased to 300 ml under manual ventilation. Since the patient had a history of asthma, we considered the possibility of an asthma attack and commenced administration of procaterol inhalation to the patient, although with no improvement. We considered interrupting the surgery, but since the surgeon stated that the operation would only require several more minutes, it was continued.

At 1 h and 34 min after commencement of the operation, not only was there elevation of EtCO_2_, but her SpO_2_ decreased to 93% and we were only able to achieve a tidal volume of approximately 50 ml with manual ventilation. Hence, we ordered the surgeon to stop the procedure.

By this time, the patient’s EtCO_2_ had reached 177 mmHg. The surgeons continued abdominal paracentesis and anesthesiologists continued manual ventilation and inserted a catheter in her radial artery for blood gas analysis (BGA). BGA indicated a pH of 7.017, PaO_2_ of 152 mmHg, PaCO_2_ of 99.3 mmHg, and B.E. of − 10.1 mmol/l under manual ventilation with 80% oxygen with air. Chest radiography showed prominent pneumomediastinum and subcutaneous emphysema. We continued manual ventilation, and the surgeon performed abdominal paracentesis again at another site for de-aeration. Subsequently, once EtCO_2_ gradually decreased over the next hour and manual ventilation became easier, we switched back to mechanical ventilation. The ventilator settings at this time were VCV with a tidal volume of 500 ml and respiratory rate of 14 breaths/min. Thereafter, PAP improved to about 15 cmH_2_O, EtCO_2_ decreased to 38 mmHg, and subcutaneous emphysema was clearly relieved. BGA at this time indicated a pH of 7.292, PaO_2_ of 91.9 mmHg, PaCO_2_ of 45.9 mmHg, and B.E. of − 4.6 mmol/l during mechanical ventilation with 50% oxygen with air.

The total interruption time was 85 min. After restarting the procedure, there was no further apparent increase in EtCO_2_ and the surgery was concluded 26 min after it was restarted. Postoperatively, we took a chest radiograph to confirm improvement of pneumomediastinum and subcutaneous emphysema. At 23 min after the end of the operation, since the patient was conscious and was breathing spontaneously with a tidal volume of about 500 ml and respiratory rate of 16 breaths/min, we extubated her trachea. BGA immediately after extubation indicated a pH of 7.319, PaO_2_ of 78.1 mmHg, PaCO_2_ of 44.5 mmHg, and B.E. of − 3.4 mmol/l under spontaneous respiration via a face mask with 5 l/min oxygen.

We transported the patient to the intensive care unit (ICU) for observation, where her respiratory status remained stable.

## Discussion

There are two important considerations for anesthesia during POEM. One is to avoid aspiration during induction of anesthesia, and the other is to be careful about the development of gas-related complications. In our case, we aimed to prevent aspiration by instructing the patient to stop eating and drinking on the night before the operation and by performing rapid sequence induction. Gas-related complications during POEM, such as subcutaneous emphysema, pneumoperitoneum, mediastinal emphysema, and pneumothorax, have been previously reported [5, 6]. Since the esophagus does not have a serosal layer, these complications can occur relatively frequently when the muscle layer is breached. Endoscopists should always be careful to avoid damaging the esophageal muscle layer during the procedure.

In our opinion, the following two reasons could account for the ventilatory impairment in this case. First, the total length of the endoscopic myotomy that was required was longer than usual because of Jackhammer esophagus. Indeed, the median total myotomy length was 14 cm in a previous report [7], whereas, in this case, the total length of the myotomy was 23 cm. This resulted in a larger amount of carbon dioxide being used during the procedure, which lead to rapidly progressive subcutaneous emphysema and pneumoperitoneum. Second, the patient’s esophageal mucosa and subcutaneous tissues might have been weak because she was on steroid therapy, which could have contributed to the rapid spread of the subcutaneous emphysema and pneumoperitoneum.

In this case, a significant respiratory acidosis of pH 7.017 and PaCO_2_ of 99.3 mmHg occurred temporarily. It is not clear how clinically acceptable the increase in PaCO_2_ or the decrease in pH was. Although we did not observe hemodynamic failure, including arrhythmia or hypotension, in our patient, we should have recommended interruption of the operation a little earlier.

## Conclusions

We report a case of unexpected ventilatory impairment during POEM under general anesthesia. Anesthesiologists should be aware that hypercarbia, pneumoperitoneum, and subcutaneous emphysema causing severe ventilatory impairment can occur during POEM for Jackhammer esophagus.

## Abbreviations

POEM: Per-oral endoscopic myotomy
